# miR-380-5p facilitates NRF2 and attenuates cerebral ischemia/reperfusion injury-induced neuronal cell death by directly targeting BACH1

**DOI:** 10.1515/tnsci-2020-0172

**Published:** 2021-05-18

**Authors:** Yibiao Wang, Min Xu

**Affiliations:** Department of Neurosurgery, Hainan Affiliated Hospital of Hainan Medical University, Haikou City, Hainan Province, 570311, China; Department of Neurosurgery, Kunshan Hospital of Traditional Chinese Medicine, Kunshan Affiliated Hospital of Nanjing University of Chinese Medicine, No. 189 Chaoyang Road, Kunshan City, Jiangsu Province, 215300, China

**Keywords:** miR-380-5p, NRF2, cerebral ischemia/reperfusion injury, neuronal cell death, BACH1

## Abstract

**Background:**

This study aimed to explore the role of miR-380-5p in cerebral ischemia/reperfusion (CIR) injury-induced neuronal cell death and the potential signaling pathway involved.

**Methodology:**

Human neuroblastoma cell line SH-SY5Y cells were used in this study. Oxygen and glucose deprivation/reperfusion (OGD/R) model was used to mimic ischemia/reperfusion injury. CCK-8 assay and flow cytometry were used to examine cell survival. Quantitative real time PCR (RT-qPCR) assay and Western blotting were used to measure the change of RNA and protein expression, respectively. TargetScan and Luciferase assay was used to confirm the target of miR-380-5p. Malondialdehyde (MDA) superoxide dismutase (SOD) and glutathione peroxidase (GSHPx) were measured using commercial kits.

**Results:**

miR-380-5p was downregulated in SH-SY5Y cells after OGD/R. Cell viability was increased by miR-380-5p, while cell apoptosis was reduced by miR-380-5p mimics. MDA was reduced by miR-380-5p mimics, while SOD and GSHPx were increased by miR-380-5p. Results of TargetScan and luciferase assay have showed that BACH1 is the direct target of miR-380-5p. Expression of NRF2 was upregulated after OGD/R, but was not affected by miR-380-5p. mRNA expression of HO-1 and NQO1 and ARE activity were increased by miR-380-5p. Overexpression of BACH1 reversed the antioxidant and neuroprotective effects of miR-380-5p.

**Conclusion:**

miR-380-5p inhibited cell death induced by CIR injury through target BACH1 which also facilitated the activation of NRF2, indicating the antioxidant and neuroprotective effects of miR-380-5p.

## 1 Introduction

Approximately 80% of strokes were caused by cerebral ischemia, which is the most common atherosclerotic disease in the cerebral blood vessels [1,2]. Cerebral ischemia/reperfusion (CIR) injury could result in serious dysfunction of brain with high risk of disability and mortality [3]. If the cerebral ischemia was not reversed within a short time, neuronal cell death would be observed around the affected vessels, termed infarction [5]. So far, there are no effective treatment for CIR injury. Thus, the therapeutic strategies of CIR injury aim to prevent neuronal cell death, which will reduce the infarct size [4].

During cerebral ischemia, the blood supply to brain was reduced, which led to the reduction of oxygen and glucose supply to the affected brain tissue [6]. Aerobic metabolism promoted the abundance of reactive oxygen species (ROS), a type of free radical, which contributed to cell necrosis and death lipid peroxidation [7]. In normal condition, ROS can be scavenged by superoxide dismutase (SOD), the most crucial antioxidant enzyme in brain [8]. SOD can convert {\text{O}}_{2}^{-}] into H_2_O_2_, and further into H_2_O and O_2_ by glutathione peroxidase (GSHPx) [7,9]. Therefore, increase of SOD and GSHPx activity could prevent cell death during CIR injury. In addition to SOD and GSHPx, nuclear factor erythroid 2-related factor 2 (NRF2) also involved into the transcription of antioxidant genes and activation of NRF2 and its target genes could prevent CIR injury in brain [10].

As a class of small noncoding RNAs, miRNAs participate in many physiopathological processes through inducing translational repression of their target mRNAs [11]. Dysregulation of miRNAs after CIR injury was observed using miRNA-profiling technique, suggesting that miRNAs engaged in the response to CIR injury [12]. Eight miRNAs have been upregulated after 3 h of reperfusion and, among them, miR-200b, miR-200c, and miR-429 have demonstrated their neuroprotective effects through targeting prolyl hydroxylase 2 [13]. The goal of this study is to figure out the dysregulation of miR-380-5p and its role in CIR injury-induced neuronal cell death, providing new insight for the treatment of CIR injury.

## 2 Methods

### 2.1 Cell culture and treatment

Human neuroblastoma cell line SH-SY5Y cells were obtained from American Type Culture Collection (ATCC, USA) and cultured into Dulbecco’s Modified Eagle’s Medium (DMEM) that contained 1% penicillin/streptomycin (Invitrogen, USA) and 10% fetal bovine serum (FBS, Gibco, USA) at 37°C with 95% air and 5% CO_2_ (normal condition) in a humidified incubator.

Oxygen and glucose deprivation/reperfusion (OGD/R) model was used to mimic ischemia/reperfusion injury in this study. Briefly, SH-SY5Y cells were cultured with glucose-free DMEM with 95% N_2_ and 5% CO_2_ (anaerobic condition) for 6 h and then cultured in normal condition for another 24 h to induce reoxygenation.

miR-380-5p mimics, miR-380-5p inhibitors, and their negative controls were obtained from Guangzhou RiboBio Co., LTD (RiboBio, China). BACH1-overexpression vectors and its control vectors were also purchased from RiboBio. 1 × 10^5^ cells per well of SH-SY5H cells were incubated in the 6 well plates. Before 48 h of OGD/R, cell transfection or co-transfection was conducted using lipofectamine 2000 according to the manufacturer’s instructions.


**Ethical approval:** The conducted research is not related to either human or animals use.

### 2.2 Examination of cell viability and apoptosis

Cell viability was detected using CCK-8 kit (Sigma-Aldrich, USA). After treated as described above, SH-SY5Y cells were seeded in the 96-well plate at the density of 10^4^ cells per well and cultured at 37°C overnight. The medium was removed, and the cells were washed with PBS. 10 µL of CCK-8 reagent was added to each well and incubated at 37°C for 2 h. The optical density value at 450 nm was measured and recorded using a Bio-Rad microplate reader.

Cell apoptosis was measured using flow cytometry. After treated as described above, SH-SY5Y cells were resuspended and stained using Annexin V-FITC Apoptosis Detection Kit (Sigma-Aldrich). Ten thousand cells were collected using flow cytometry (FACScan, USA) and the results were analyzed using FlowJo^TM^ v10.6.1 (FlowJo, USA).

### 2.3 Measurement of malondialdehyde (MDA), SOD, GSHPx, and antioxidant response element (ARE)

Change of MDA, SDO, GSHPx, and ARE was measured using the commercial kits, Lipid Peroxidation (MDA) Assay Kit (Sigma-Aldrich), Superoxide Dismutase Activity Assay Kit (Abcam, United Kingdom), Glutathione Peroxidase Assay Kit (Cayman Chemicals, USA), and ARE Reporter Kit (Bioscience, USA), respectively, according to the manufacturer’s protocols.

### 2.4 Luciferase assay

The 3′ untranslated region (3′-UTR) of BACH1 and its mutant sequence (GeneCopoeia, USA) were inserted into the pGL3 vector (Promega, USA) to form luciferase reporter plasmids, BACH1-WT and BACH1-MUT, respectively. miR-380-5p mimics, miR-380-5p inhibitors or their negative controls, were then co-transfected with BACH1-WT and BACH1-MUT, respectively. The change of luciferase activity was measured using the Promega Dual-Luciferase Reporter Assay System.

### 2.5 RNA extract and quantitative real time PCR (RT-qPCR) assay

Total RNA was extracted using PureLink^TM^ RNA Mini Kit (Thermo Fisher, USA). 10 ng of RNA was used to reverse transcription to synthesize cDNA using QuantiTect Reverse Transcription Kit (QIAGEN, Germany), and RT-qPCR was performed using QIAGEN OneStep RT-PCR Kit (QIAGEN). RT-qPCR of miR-380-5p was performed using miScript SYBR^®^ Green PCR Kit (QIAGEN). Relative expression of RNAs was quantified using 2^−ΔΔCT^ method [14]. The primers used in this study were: U6 forward 5′-CTCGCTTCGGCAGCACA-3′ and reverse 5′-AACGCTTCACGAATTTGCGT-3′; HO-1, forward 5′-ATGACACCAAGGACCAGAGC-3′ and reverse 5′-TGTAAGGACCCATCGGAGA-3′; NQO1, forward 5′-CTCGCCTCATGCGTTTTTG-3′ and reverse 5′-CCCCTAATCTGACCTCGTTCAT-3′; β-actin forward, 5′-AGCCTCGCCTTTGCCGA-3′ and reverse, 5′-CTGGTGCCTGGGGCG-3′.

### 2.6 Western blotting

The treated SH-SY5Y cells were lysed to extract the proteins using RIPA lysis and extraction buffer (Thermo Fisher). Nuclear proteins were extracted using Cytoplasmic And Nuclear Protein Extraction Kit (BosterBio, USA). 5 µg of total protein was loaded into 8% of sodium dodecyl sulphate-polyacrylamide gel and separated by electrophoresis. The separated protein was transferred from the gels to the PVDF membranes and the PVDF membranes were blocked by 5% of fat-free milk for 1 h at room temperature. The proper primary antibodies were used to probe the target proteins overnight at 4°C followed by incubating the membrane with proper secondary antibodies (ThermoFisher) for 2 h at room temperature. The strength of protein signal was detected using SignalFire™ ECL Reagent (CST, USA). The primary antibodies used in this study were: BACH1 (sc-271211, 1:1,000 dilution), NRF2 (ab137550, 1:1,000 dilution), Lamin B2 (ab233530, 1:800 dilution), and β-actin (ab8227, 1:5,000 dilution).

### 2.7 Statistical analysis

All statistical analyses were performed using SPSS v19.0 (IBM, USA). All experiment data were presented as mean ± standard error of mean (SEM). Student’s *t* test and one-way ANOVA were used to compare the difference between two groups and multiple groups, respectively. *p* value less than 0.05 was considered statistically significant difference.

## 3 Results

### 3.1 Upregulation of miR-380-5p promoted cell survival and inhibited cell apoptosis induced by OGD/R

**Figure 1:**
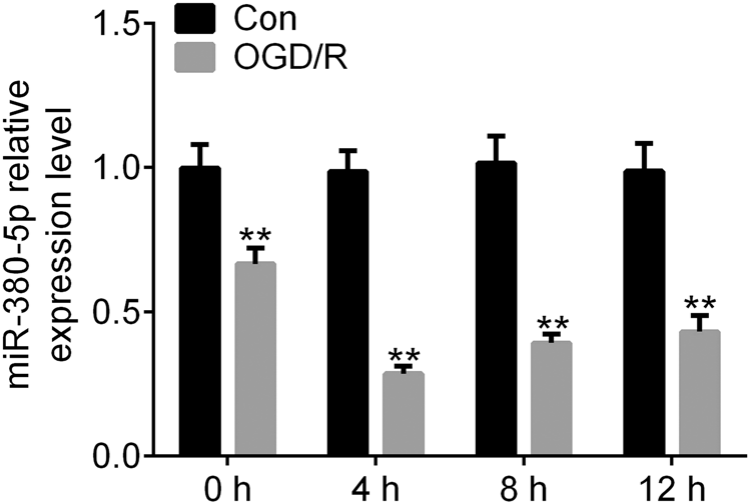
Expression of miR-380-5p was downregulated by OGD/R. **p < 0.01 vs Con. Con: Control; OGD/R: oxygen and glucose deprivation/reperfusion.

**Figure 2:**
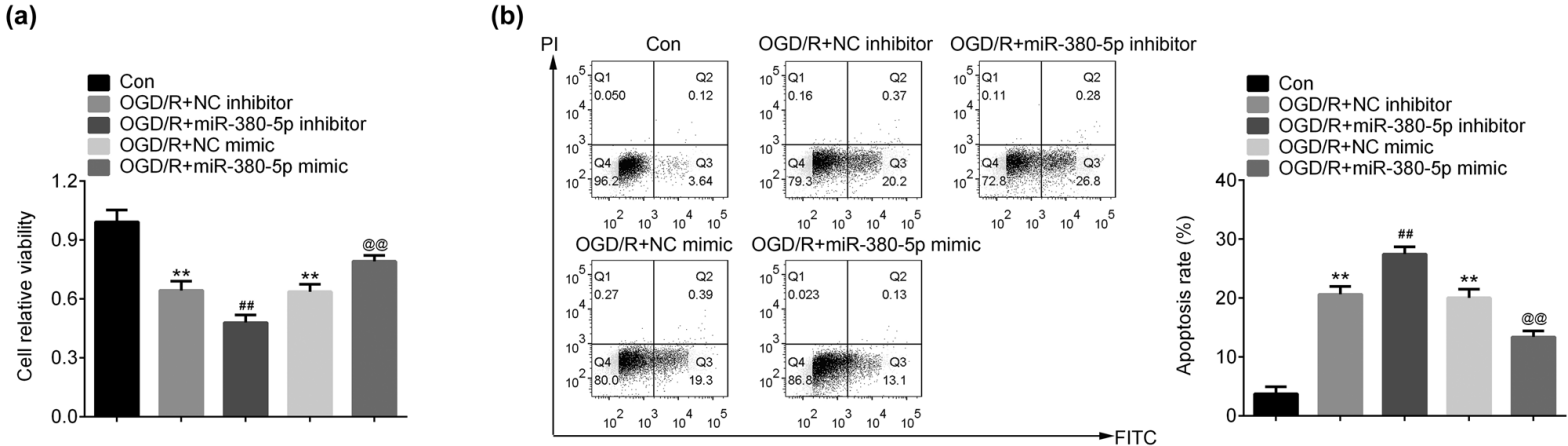
Upregulation of miR-380-5p promoted cell survival and inhibited cell apoptosis induced by OGD/R. (a) miR-380-5p prevented the reduction of cell viability induced by OGD/R. (b) miR-380-5p inhibited cell apoptosis induced by OGD/R. **p < 0.01 vs Con; ^##^ *p* < 0.01 vs NC inhibitor; ^@@^ *p* < 0.01 vs NC mimics. NC: negative inhibitor.

Expression of miR-380-5p was significantly downregulated at 4, 8, and 12 h after OGD/R in SH-SY5Y cells (Figure 1). Cell viability was greatly reduced after OGD/R (Figure 2a). The reduction of cell viability was further enhanced by inhibition of miR-380-5p and was reversed by overexpression of miR-380-5p (Figure 2a). The number of apoptotic cells was significantly increased after OGD/R (Figure 2b). Cell apoptosis was increased by miR-380-5p inhibitors and reduced by miR-380-5p mimics after OGD/R (Figure 2b).

### 3.2 Overexpression of miR-380-5p repressed the oxidative stress after OGD/R

**Figure 3:**
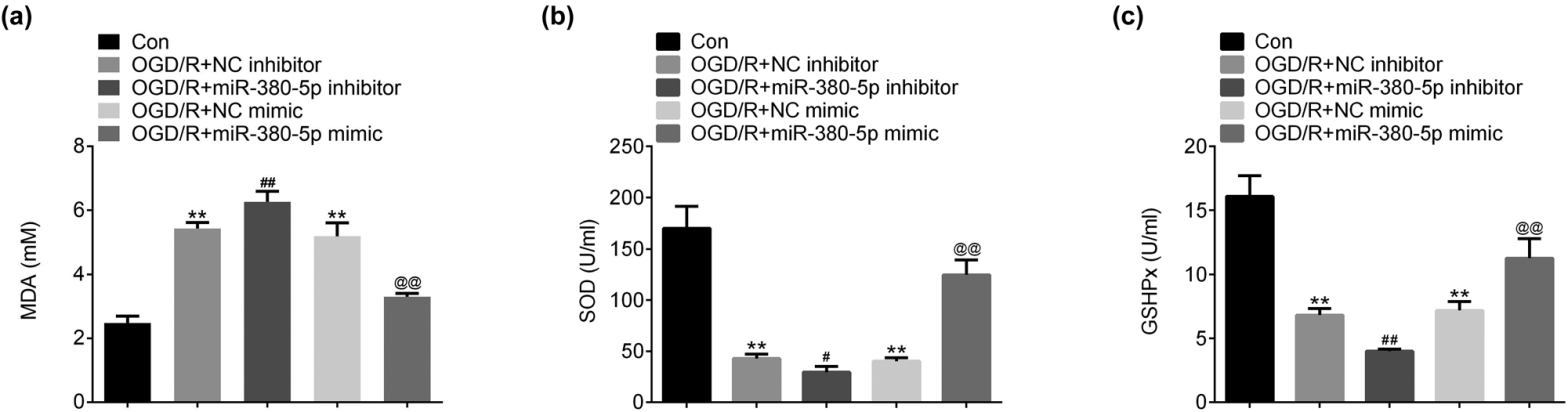
Overexpression of miR-380-5p repressed the oxidative stress after OGD/R. (a) miR-380-5p inhibited OGD/R-induced increase of MDA. (b) miR-380-5p inhibited OGD/R-induced reduction of SOD. (c) miR-380-5p inhibited OGD/R-induced reduction of GSHPx. **p < 0.01 vs Con; ^##^ *p* < 0.01 vs NC inhibitor; ^@@^ *p* < 0.01 vs NC mimics. MDA: malondialdehyde; SOD: superoxide dismutase; GSHPx: glutathione peroxidase.

After OGD/R, MDA level was increased in SH-SY5Y cells (Figure 3a). MDA level was further increased after OGD/R in cells transfected with miR-380-5p inhibitors compared with control group, while the OGD/R-induced increase of MDA was inhibited in cells transfected with miR-380-5p mimics compared with control group (Figure 3a). After OGD/R, SOD was significantly reduced in SH-SY5Y cells (Figure 3b). The reduction of SOD induced by OGD/R was promoted by miR-380-5p inhibitors and prevented by miR-380-5p mimics (Figure 3b). Similarly, GSHPx was significantly reduced after OGD/R in SH-SY5Y cells (Figure 3c). The reduction of GSHPx induced by OGD/R was promoted by miR-380-5p inhibitors and prevented by miR-380-5p mimics (Figure 3c).

### 3.3 miR-380-5p directly targeted the 3′-UTR of BACH1

**Figure 4:**
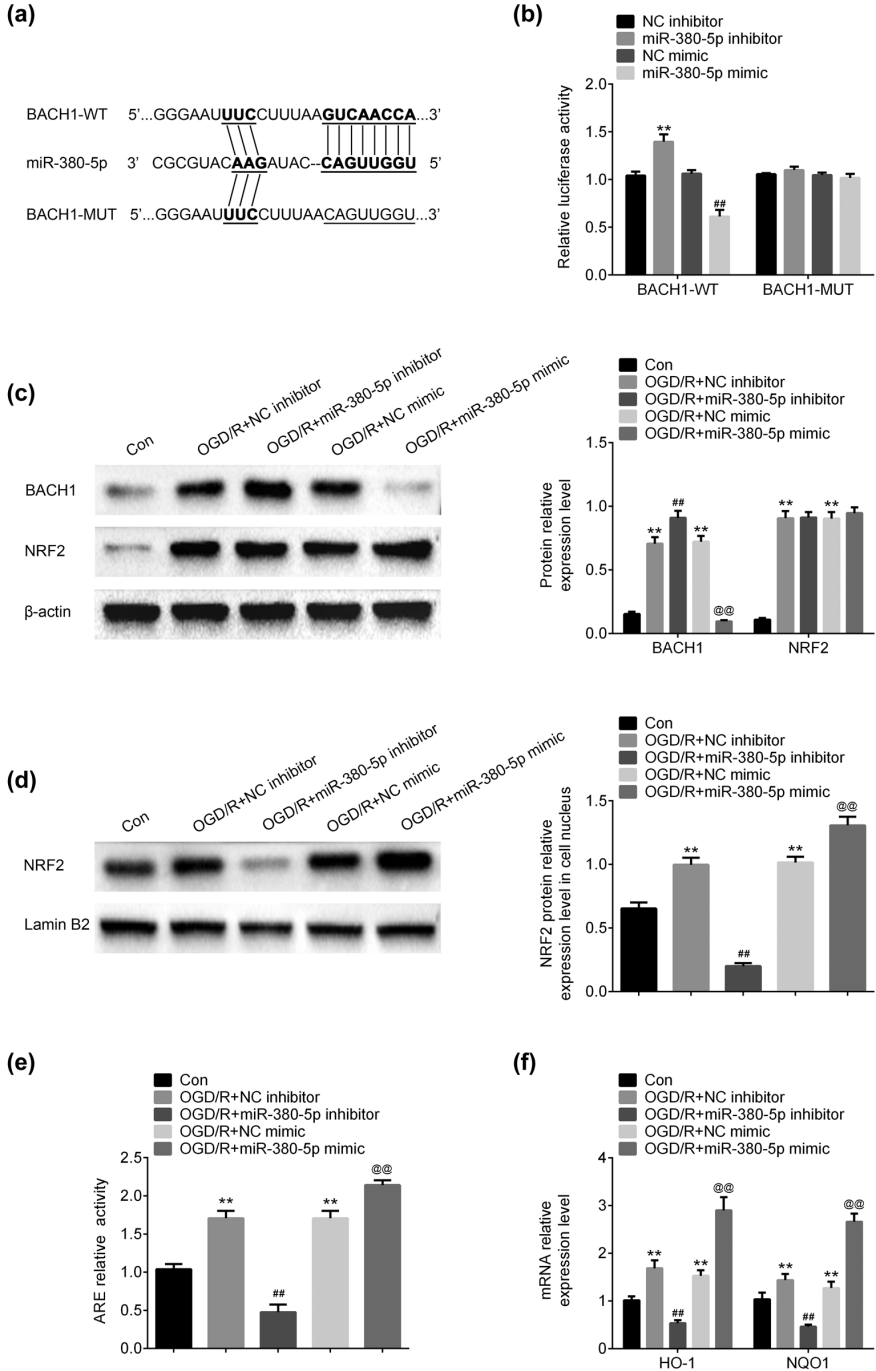
miR-380-5p directly targeted the 3′-UTR of BACH1. (a) A complementary sequence was observed between 3′-UTR of BACH1 and miR-380-5p. (b) Luciferase activity was increased by miR-380-5p inhibitors and decreased by miR-380-5p mimics in BACH1-WT group. (c) Expression of BACH1 was downregulated by miR-380-5p mimics, while expression of NRF2 did not show any significant difference. (d) Expression of BACH1 was upregulated by miR-380-5p mimics in cell nucleus. (e) miR-380-5p promoted the increase of ARE activity induced by OGD/R. (f) mRNA expression of HO-1 and NQO1 was upregulated by miR-380-5p after OGD/R. **p < 0.01 vs Con; ^##^ *p* < 0.01 vs NC inhibitor; ^@@^ *p* < 0.01 vs NC mimics.

TargetScan (www.targetscan.org) was used to predict the potential target of miR-380-5p. The results demonstrated that there was a complementary sequence between 3′-UTR of BACH1 and miR-380-5p (Figure 4a). Luciferase assay showed that the luciferase activity was increased by miR-380-5p inhibitors and reduced by miR-308-5p in cell co-transfected with BACH1-WT, while the luciferase activity did not show any significant change in cells co-transfected with BACH1-MUT (Figure 4b). After OGD/R, both BACH1 and NRF2 expressions were upregulated in SH-SY5Y cells (Figure 4c). The expression of BACH1 was upregulated by miR-380-5p inhibitors and downregulated by miR-380-5p mimics compared with negative control groups (Figure 4c). The expression of NRF2 was not affected by either miR-380-5p inhibitors or miR-380-5p mimics (Figure 4c). In cell nucleus, overexpression of NRF2 induced by OGD/R was downregulated by miR-380-5p inhibitors and upregulated by miR-380-5p mimics (Figure 4d). The relative activity of ARE was increased after OGD/R (Figure 4e). The increased activity of ARE was inhibited by miR-380-5p inhibitors and induced by miR-380-5p mimics (Figure 4e). The mRNA level of HO-1 and NQO1 was upregulated after OGD/R (Figure 4f). Inhibition of miR-380-5p downregulated HO-1 and NQO1 mRNA expression and overexpression of miR-380-5p upregulated HO-1 and NQO1 mRNA expression (Figure 4f)

### 3.4 Overexpression of BACH1 reversed miR-380-5p-mediated neuroprotection on SH-SY5Y cells

**Figure 5:**
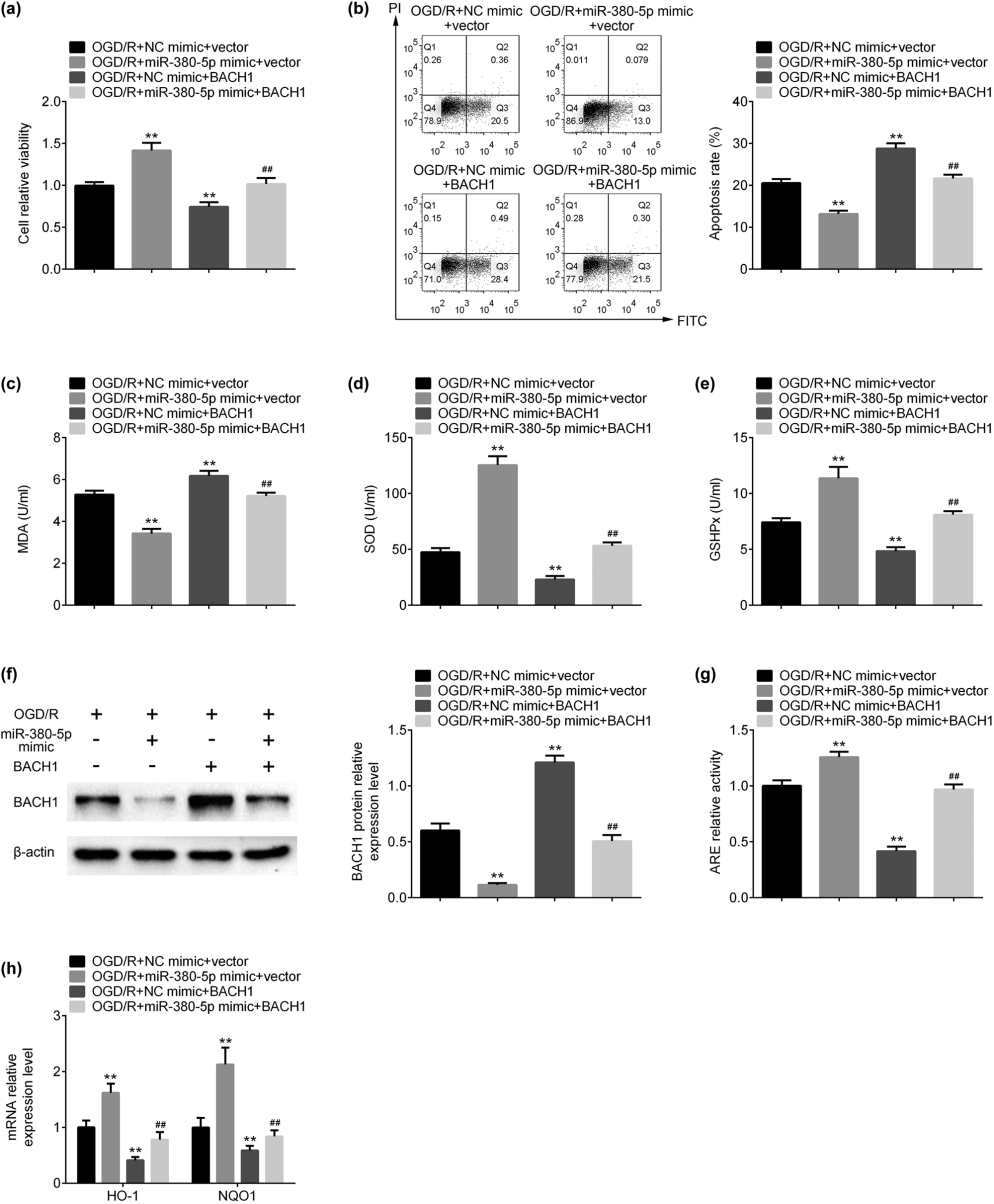
Overexpression of BACH1 reversed miR-380-5p-mediated neuroprotection on SH-SY5Y cells. (a) Overexpression of BACH1 inhibited the increase of cell viability induced by miR-380-5p mimics. (b) Overexpression of BACH1 inhibited the reduction of cell apoptosis induced by miR-380-5p mimics. (c) Overexpression of BACH1 inhibited the reduction of MDA induced by miR-380-5p mimics. (d) Overexpression of BACH1 inhibited the increase of SOD induced by miR-380-5p mimics. (e) Overexpression of BACH1 inhibited the increase of GSHPx induced by miR-380-5p mimics. (f) Overexpression of BACH1 inhibited the downregulation of BACH1 expression induced by miR-380-5p mimics. (g) Overexpression of BACH1 inhibited the increase of ARE activity induced by miR-380-5p mimics. (h) Overexpression of BACH1 inhibited the upregulation of HO-1 and NQO1 mRNA expression induced by miR-380-5p mimics. **p < 0.01 vs OGD/R + NC mimic + vector; ^##^ *p* < 0.01 vs OGD/R + miR-380-5p mimic + vector.

Cell viability was increased by miR-380-5p mimics and reduced by overexpression of BACH1 in SH-SY5H cells after OGD/R (Figure 5a). Cell apoptosis was reduced by miR-380-5p mimics and induced by overexpression of BACH1 in SH-SY5H cells (Figure 5b). The increase of cell viability and the reduction of cell apoptosis induced by miR-380-5p mimics were inhibited by dual-upregulation of miR-380-5p and BACH1 (Figure 5a and b). MDA level was decreased by miR-380-5p mimics and increased by BACH1 vectors (Figure 5c). miR-380-5p-induced reduction of MDA was prevented by co-transfection of miR-380-5p and BACH1 (Figure 5c). SOD and GSHPx were increased by miR-380-5p mimics and reduced by BACH1 vectors (Figure 5d and e). Induction of SOD and GSHPx by miR-380-5p mimics was prevented by co-transfection of miR-380-5p and BACH1 (Figure 5d and e). Protein expression of BACH1 was downregulated by miR-380-5p mimics and upregulated by BACH1 vectors (Figure 5f). Inhibition of BACH1 by miR-380-5p mimics was reversed by co-transfection of miR-380-5p and BACH1 (Figure 5f). ARE activity, heme oxygenase-1 (HO-1), and NADPH quinone oxidoreductase 1 (NQO1) mRNA expression were increased by miR-380-5p mimics and decreased by BACH1 vectors (Figure 5g and h). Dual-upregulation of miR-380-5p and BACH1 inhibited the reduction of ARE activity, HO-1, and NQO1 mRNA expression by miR-380-5p mimics (Figure 5g and h).

## 4 Discussion

As mentioned in the introduction part, CIR injury could result in serious dysfunction of brain with high risk of disability and mortality and there is no effective treatment for CIR injury [4]. Thus, a better understanding of the mechanism of CIR injury may help to provide the new therapeutic strategy for CIR patients. In this study, expression of miR-380-5p was repressed after OGD/R. Further experiment demonstrated that miR-380-5p could attenuate the neuroblastoma cell death induced by oxidative stress after CIR injury and this process was medicated by direct targeting BACH1 and facilitating NRF2, indicating that miR-380-5p played the neuroprotective role during CIR injury.

Dysregulation of miRNAs has been found in many pathological pathways [11]. Previous study has found that high expression of miR-380-5p promoted cellular survival through target 3′-UTR of p53 in neuroblastoma model, resulting in the poor outcomes of neuroblastoma [15]. Similarly, data of this study have demonstrated that miR-380-5p also contributed to neuroblastoma cell survival after OGD/R. But, in this case, miR-380-5p targeted 3′-UTR of BACH1 to protect brain dysfunction after CIR injury. By heterodimerizing with Maf proteins, BACH1 could inhibit the transcription of HO-1 and NQO1, 2 oxidative stress-response genes, by binding to Maf recognition elements (MAREs) [16]. In this study, both inhibition of miR-380-5p and overexpression of BACH1 have downregulated the mRNA expression of HO-1 and NQO1, in accordance with previous findings. Furthermore, overexpression of BACH1 reversed the neuroprotective effects of miR-380-5p after CIR injury, proving that BACH1 is the direct target of miR-380-5p.

BACH1 and NRF2 are competitors for binding to MAREs in response to oxidative stress [16]. During oxidative stress, NRF2 is activated and releases from Kelch-like ECH-associated protein 1 (Keap1), and translocates into the nucleus, where it competitively binds to MAREs, initiating the transcription of HO-1 and NQO1 [17,18]. Data of this study have showed that expression of NRF2 was upregulated after OGD/R, but was not affected by miR-380-5p inhibitors of mimics. However, NRF2 expression in nucleus was increased by miR-380-5p mimic. These data indicated that upregulation of miR-380-5p facilitated the activation of NRF2-Keap1 signaling pathway through repression of BACH1.

After heterodimerizing with MAREs, the target genes of NRF2, called antioxidant response elements (ARE), were also activated [19]. ARE activity was increased by miR-380-5p mimics and decreased by miR-380-5p inhibitors, implying the antioxidant effects of miR-380-5p in neuroblastoma cells after CIR injury. MDA is the terminal production of lipid peroxidation and used to measure the level of lipid peroxidation [20]. As mentioned, SOD and GHSPx are two antioxidant enzymes [7]. Results of this study have manifested that miR-380-5p inhibited MDA level and increased SOD and GHSPx levels, suggesting that miR-380-5p played an antioxidant role in CIR injury, which prevented neuronal cell injury induced by OGD/R.

In conclusion, data of this study have showed that expression of miR-380-5p was repressed after OGD/R. Further experiment demonstrated that miR-380-5p could attenuate the neuroblastoma cell death induced by oxidative stress after CIR injury and this process was medicated by direct targeting BACH1 and facilitating NRF2, indicating that miR-380-5p played the neuroprotective role during CIR injury. These results provide the new insight to CIR injury.

## References

[1] Hasso AN, Stringer WA, Brown KD. Cerebral ischemia and infarction. Neuroimaging Clin N Am. 1994 Nov;4(4):733–52. 7858918

[2] Lapi D, Colantuoni A. Remodeling of cerebral microcirculation after ischemia-reperfusion. J Vasc Res. 2015;52(1):22–31. 10.1159/000381096 25896412

[3] Schmidt JM. Heart rate variability for the early detection of delayed cerebral ischemia. J Clin Neurophysiol. 2016 Jun;33(3):268–74. 10.1097/WNP.0000000000000286 27258451

[4] Ishida A, Kawakami H, Yasuzumi F, Morishita R. Gene therapy for cerebral infarction (cerebral ischemia). No Shinkei. 2002 Mar;54(3):213–9. 11968812

[5] Schaller B, Graf R. Cerebral ischemia and reperfusion: the pathophysiologic concept as a basis for clinical therapy. J Cereb Blood Flow Metab. 2004 Apr;24(4):351–71. 10.1097/00004647-200404000-00001 15087705

[6] Yang J, Chen M, Cao RY, Li Q, Zhu F. The role of circular RNAs in cerebral ischemic diseases: ischemic stroke and cerebral ischemia/reperfusion injury. Adv Exp Med Biol. 2018;1087:309–25. 10.1007/978-981-13-1426-1_25 30259377

[7] Chen CH, Hsieh CL. Effect of acupuncture on oxidative stress induced by cerebral ischemia-reperfusion Injury. Antioxid (Basel). 2020 Mar;9(3):248. 10.3390/antiox9030248 PMC7139408 32204376

[8] Wang Y, Branicky R, Noë A, Hekimi S. Superoxide dismutases: dual roles in controlling ROS damage and regulating ROS signaling. J Cell Biol. 2018 Jun;217(6):1915–28. 10.1083/jcb.201708007 PMC5987716 29669742

[9] Taysi S, Tascan AS, Ugur MG, Demir M. Radicals, oxidative/nitrosative stress and preeclampsia. Mini Rev Med Chem. 2019;19(3):178–93. 10.2174/1389557518666181015151350 30324879

[10] Zhang R, Xu M, Wang Y, Xie F, Zhang G, Qin X. Nrf2-a promising therapeutic target for defensing against oxidative stress in stroke. Mol Neurobiol. 2017 Oct;54(8):6006–17. 10.1007/s12035-016-0111-0 27696223

[11] Zhang Y, Sun X, Icli B, Feinberg MW. Emerging roles for microRNAs in diabetic microvascular disease: novel targets for therapy. Endocr Rev. 2017 Apr;38(2):145–68. 10.1210/er.2016-1122 PMC5460677 28323921

[12] Ouyang YB, Stary CM, Yang GY, Giffard R. microRNAs: innovative targets for cerebral ischemia and stroke. Curr Drug Targets. 2013 Jan;14(1):90–101. 10.2174/138945013804806424 PMC3673881 23170800

[13] Lee ST, Chu K, Jung KH, Yoon HJ, Jeon D, Kang KM, et al. MicroRNAs induced during ischemic preconditioning. Stroke. 2010 Aug;41(8):1646–51. 10.1161/STROKEAHA.110.579649 20576953

[14] Livak KJ, Schmittgen TD. Analysis of relative gene expression data using real-time quantitative PCR and the 2(-Delta Delta C(T)) method. Methods. 2001 Dec;25(4):402–8. 10.1006/meth.2001.1262 11846609

[15] Swarbrick A, Woods SL, Shaw A, Balakrishnan A, Phua Y, Nguyen A, et al. miR-380-5p represses p53 to control cellular survival and is associated with poor outcome in MYCN-amplified neuroblastoma. Nat Med. 2010 Oct;16(10):1134–40. 10.1038/nm.2227 PMC3019350 20871609

[16] Zhang X, Guo J, Wei X, Niu C, Jia M, Li Q, et al. Bach1: function, regulation, and involvement in disease. Oxid Med Cell Longev. 2018 Oct;2018:1347969. 10.1155/2018/1347969 PMC6189649 30370001

[17] Mizumura K, Maruoka S, Shimizu T, Gon Y. Role of Nrf2 in the pathogenesis of respiratory diseases. Respir Investig. 2020 Jan;58(1):28–35. 10.1016/j.resinv.2019.10.003 31704266

[18] Igarashi K, Sun J. The heme-Bach1 pathway in the regulation of oxidative stress response and erythroid differentiation. Antioxid Redox Signal. 2006 Jan-Feb;8(1–2):107–18. 10.1089/ars.2006.8.107 16487043

[19] Buendia I, Michalska P, Navarro E, Gameiro I, Egea J, León R. Nrf2-ARE pathway: an emerging target against oxidative stress and neuroinflammation in neurodegenerative diseases. Pharmacol Ther. 2016 Jan;157:84–104. 10.1016/j.pharmthera.2015.11.003 26617217

[20] Zhong H, Hao L, Li X, Wang C, Wu X. Anti-inflammatory role of trilobatin on lipopolysaccharide-induced acute lung injury through activation of AMPK/GSK3β-Nrf2 pathway. Signa Vitae. 2020 Oct;16(2):160–6.

